# Oxidative Stress in Pathogenesis of Preeclampsia: Mechanistic and Clinical Insights

**DOI:** 10.3390/antiox15030387

**Published:** 2026-03-19

**Authors:** Natnipa Parapob, Suchaya Luewan, Threebhorn Kamlungkuea, Theera Tongsong

**Affiliations:** 1 Fetal Center, Faculty of Medicine, Chiang Mai University, Chiang Mai 50200, Thailand; natnipa.p@cmu.ac.th (N.P.);; 2 Department of Obstetrics and Gynecology, Chiang Mai University, Chiang Mai 50200, Thailand

**Keywords:** antioxidant, endothelial dysfunction, oxidative stress, placenta, preeclampsia, reactive oxygen species, trophoblast

## Abstract

Preeclampsia, affecting 3–8% of pregnancies worldwide, remains a leading cause of maternal and perinatal morbidity and mortality. This review synthesizes current molecular, immunological, and hemodynamic evidence to clarify the central role of oxidative stress in the pathogenesis of preeclampsia. Placental oxidative stress, resulting from an imbalance between reactive oxygen species (ROS) generation and antioxidant defenses, secondary to placental hypoxia due to various etiologies especially impaired spiral artery remodeling, drives mitochondrial dysfunction in trophoblasts, ischemia–reperfusion injury, inflammatory pathway activation, and disruption of angiogenic homeostasis, thereby promoting systemic inflammation. Key regulatory pathways, including Nrf2/HO-1, NF-κB, PI3K/Akt, and HIF-1α, together with biomarkers such as malondialdehyde, 8-isoprostane, and the sFlt-1/PlGF ratio, characterize this redox imbalance. Although experimental studies demonstrate promising effects of targeted antioxidants, mitochondria-directed agents, and pathway-specific modulators, clinical translation remains limited, as non-specific antioxidants such as vitamins C and E have failed to prevent preeclampsia. Future advances will likely depend on mechanism-based therapies initiated early in pregnancy and tailored to the disease subtype and biomarker profiles. Collectively, this review provides an integrated mechanistic framework and highlights critical knowledge gaps that must be addressed to enable the development of effective preventive and therapeutic interventions for preeclampsia.

## 1. Introduction

Preeclampsia is a multisystem hypertensive disorder of pregnancy characterized by new-onset hypertension (≥140/90 mmHg) after 20 weeks of gestation, commonly accompanied by proteinuria and maternal organ dysfunction or uteroplacental impairment [1,2]. Affecting approximately 3–8% of pregnancies worldwide, it remains a leading cause of maternal and perinatal morbidity and mortality, accounting for over 70,000 maternal deaths annually [3,4,5]. Despite extensive investigation, the precise pathogenesis of preeclampsia has not been fully elucidated. Increasing evidence over recent decades implicates oxidative stress as a central contributor to disease initiation and progression [6,7,8]. Oxidative stress arises when reactive oxygen species (ROS) production exceeds the neutralizing capacity of antioxidant defense systems [6,9,10].

In preeclampsia, redox imbalance is evident within the placenta and maternal vascular endothelium, promoting endothelial dysfunction, exaggerated inflammatory responses, and impaired placental perfusion [11,12]. The oxidative stress model proposes that abnormal placentation results in intermittent placental hypoxia–reoxygenation injury, leading to excessive ROS generation that overwhelms maternal antioxidant defenses [6,10,13,14]. This pro-oxidant milieu induces lipid and protein peroxidation, oxidative DNA damage, and activation of pro-inflammatory signaling pathways, ultimately driving the clinical manifestations of preeclampsia, including hypertension, proteinuria, and multisystem organ involvement [15,16].

This comprehensive review synthesizes current evidence on oxidative stress in preeclampsia, focusing on molecular mechanisms, clinically relevant biomarkers, and emerging therapeutic approaches. It integrates findings from 160 peer-reviewed studies mainly published between 2014 and 2025, highlighting recent advances in understanding placental hypoxia, mitochondrial dysfunction, dysregulated signaling pathways, and potential novel therapeutic targets.

## 2. Sources and Selection Criteria

A comprehensive electronic search was conducted in PubMed, Scopus, and Web of Science using the keywords “(pre-eclampsia OR preeclampsia) AND (placenta OR placental) AND (oxidative stress)” through December 2025 without date restrictions. The search yielded 1347 citations. Approximately one-third of the included references were published before 2014, while the remainder were published within the past decade, with the most recent appearing in 2025. Only English-language articles were considered. Additional relevant studies were identified through manual screening of reference lists by the senior author (TT), and they were quality-assessed, validated and synthesized by the author’s team. Eligible studies included peer-reviewed, data-driven research, primarily clinical studies, animal experiments, in vitro investigations, and meta-analyses, relevant to three themes: (1) molecular mechanisms by which oxidative stress contributes to the development of preeclampsia; (2) major signaling pathways and associated biomarkers; and (3) therapeutic strategies targeting oxidative stress. Studies not aligned with these themes were excluded. The selected studies were manually reviewed and synthesized to address the objectives of this review. In total, evidence from 160 peer-reviewed publications was integrated, with priority given to studies elucidating the mechanisms of oxidative stress in preeclampsia as well as clinical associations. Human studies were emphasized when available, supplemented by findings from animal models and in vitro investigations. Earlier studies and reviews were cited selectively to establish foundational concepts in the pathogenesis of preeclampsia.

## 3. Background

### 3.1. Uterine Artery Remodeling

**Figure 1:**
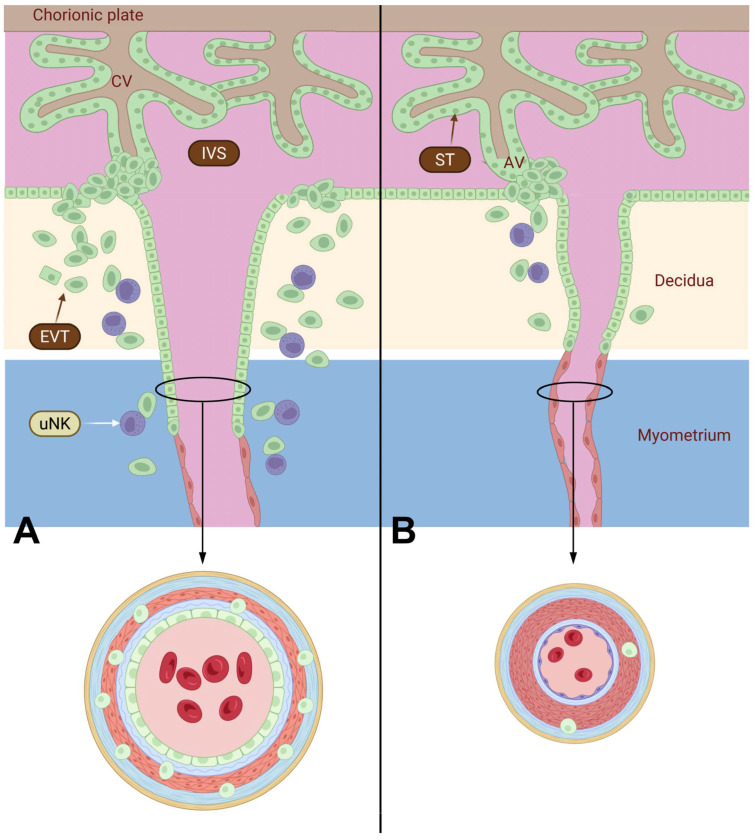
(**A**) *Normal remodeling*: Extravillous trophoblasts (EVTs) originating from anchoring villi invade the decidua and extend into the myometrium. Endovascular EVTs infiltrate the spiral arteries, replacing the endothelial lining and penetrating deeply into the decidual and partial myometrial segments. These cells release angiogenic mediators that promote marked vasodilation. Interstitial EVTs, together with uterine natural killer (uNK) cells, remodel the connective tissue, resulting in reduced surrounding resistance and decreased muscular tone, thereby enabling further arterial dilation and structural adaptation. Successful remodeling is maintained under conditions of redox balance within a milieu of low-to-moderate reactive oxygen species (ROS) levels. (**B**) *Abnormal remodeling:* EVTs incompletely replace the endothelial lining and fail to penetrate deeply into the myometrial layer. Inadequate vascular transformation leads to insufficient placental perfusion, causing relative ischemia and subsequent hypoxia–reperfusion injury. This process generates excessive local ROS, impairing both vascular and extracellular matrix remodeling. (IVS: intervillous space; ROS: reactive oxygen species; ST: syncytiotrophoblast; CV: chorionic villi; AV: anchoring villi). Created in Biorender. Theera Tongsong (2026), https://app.biorender.com/illustrations/698fd08182d9d3bd9b994390 (accessed on 27 February 2026).

During normal pregnancy, uteroplacental blood flow increases markedly to meet fetal demands, accompanied by expansion of the pelvic vasculature and extensive uterine arterial remodeling. This tightly regulated, multistep process is primarily mediated by extravillous trophoblasts (EVTs) and involves vascular enlargement and elongation through endothelial and smooth muscle cell hypertrophy and hyperplasia, together with extracellular matrix (ECM) reorganization [17]. Trophoblasts differentiate into villous and extravillous subtypes: villous trophoblasts form cytotrophoblasts and syncytiotrophoblasts that line the placental villi, whereas EVTs invade the decidua and inner myometrium early in gestation [18,19]. EVTs remodel spiral arteries by replacing the endothelium and disrupting the musculoelastic layer with fibrinoid material, thereby transforming them into dilated, low-resistance vessels capable of sustaining adequate placental perfusion (Figure 1). This remodeling and vascular branching are regulated by angiogenic mediators, including nitric oxide (NO), vascular endothelial growth factor (VEGF), placental growth factor (PlGF), soluble fms-like tyrosine kinase-1 (sFlt-1), soluble endoglin (sEng), and angiopoietins (Ang-1 and Ang-2).

In spiral arteries, endothelial replacement by EVTs generates high-capacitance vessels largely independent of maternal vasomotor control [20]. Uterine vascular tone concurrently declines through endothelial reprogramming characterized by increased endothelial nitric oxide synthase (eNOS) activity, reduced endothelin-1 signaling, and enhanced endothelium-derived hyperpolarizing factor (EDHF)-mediated relaxation. These adaptations involve coordinated regulation of matrix metalloproteinases (MMPs), tissue inhibitors of metalloproteinases (TIMP-2), and membrane-type 1 MMP, while vascular smooth muscle cells shift from a contractile state to a synthetic phenotype, increasing MMP secretion to permit controlled extracellular matrix (ECM) remodeling and luminal expansion [21,22]. These structural changes are further supported by angiogenic factors and nitric oxide within a physiological redox environment that promotes eNOS phosphorylation, VEGF signaling, and redox-sensitive MMP activation [23].

**Figure 2:**
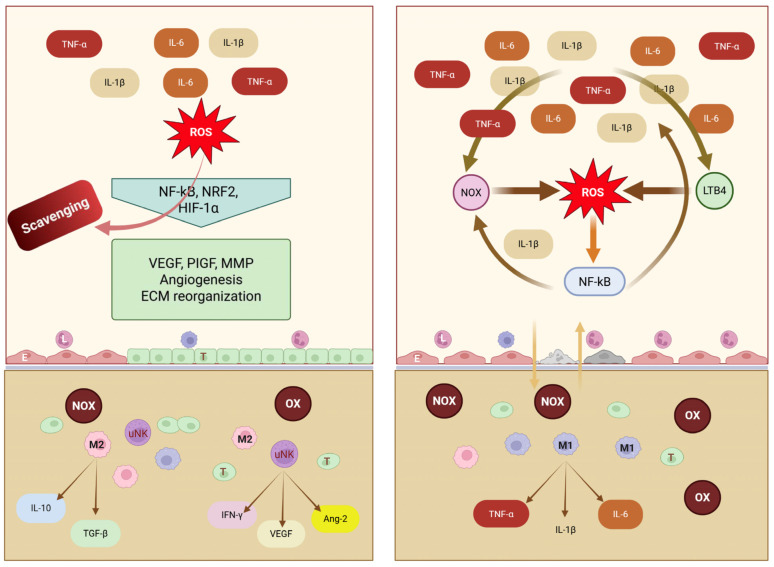
*Pro-inflammatory responses in spiral artery remodeling*: Trophoblasts (T) and macrophages (M1 and M2) secrete cytokines (e.g., IL-1β, TNF-α, and IL-6) that upregulate endothelial adhesion molecules (VCAM-1 and ICAM-1) and promote leukocyte recruitment. (**Left**): *Normal placenta*. Uterine natural killer (uNK) cells produce IFN-γ, VEGF, and angiopoietin-2, facilitating immune tolerance and spiral artery transformation. Predominantly anti-inflammatory M2 macrophages release TGF-β, supporting extracellular matrix remodeling and vasodilation. Moderate levels of ROS activate redox-sensitive transcription factors (NF-κB, Nrf2, and HIF-1α) and induce the expression of pro-angiogenic and matrix-remodeling mediators, such as VEGF, PlGF, and matrix metalloproteinases (MMPs), thereby promoting angiogenesis, extracellular matrix remodeling, and vasodilation. (**Right**): *Preeclamptic placenta*. Excessive ROS results in more inflammatory responses. Pro-inflammatory M1 macrophages predominate. Cytokine-driven activation of NADPH oxidase (NOX) increases ROS, resulting in eNOS downregulation, increased endothelial permeability, and enhanced leukocyte recruitment. M1-derived TNF-α and IL-1β further amplify oxidative stress and endothelial dysfunction, impairing spiral artery remodeling. Trophoblast-derived leukotrienes additionally disrupt endothelial integrity and vascular adaptation. (ECM: extracellular matrix; HIF-1α: hypoxia-inducible factor 1-alpha; MMP: matrix metalloproteinase; NF-κB: nuclear factor kappa B; NOX: NADPH oxidase; NRF2: nuclear factor erythroid 2-like 2; PlGF: placental growth factor; VEGF: vascular endothelial growth factor; OX: oxidase xanthine). Created in Biorender. Theera Tongsong (2026), https://app.biorender.com/illustrations/698fd08182d9d3bd9b994390 (accessed on 27 February 2026).

Uterine natural killer (uNK) cells, the predominant decidual leukocyte subset, together with macrophages, are essential regulators of uterine vascular remodeling (Figure 2) [20,24]. Unlike peripheral NK cells, uNK cells exhibit low cytotoxicity and instead secrete cytokines and angiogenic mediators that regulate trophoblast invasion, spiral artery transformation, and clearance of cellular debris [25,26,27,28]. Through reciprocal chemokine signaling with trophoblasts, uNK cells promote vascular adaptation via secretion of interferon-γ, VEGF, angiopoietin-2, and PlGF, which support vasodilation, ECM remodeling, and immune tolerance toward semi-allogeneic trophoblasts [29,30]. Oxidative stress in preeclampsia may impair uNK cell function, contributing to defective vascular remodeling and reduced placental perfusion.

Decidual macrophages are broadly classified into pro-inflammatory M1 and immunoregulatory M2 phenotypes [30,31,32]. M1 macrophages produce IL-1β, TNF-α, and IL-6, whereas M2 macrophages promote trophoblast invasion and vascular remodeling through anti-inflammatory mediators (IL-10 and TGF-β) and MMP-mediated ECM degradation. In preeclampsia, macrophage polarization shifts toward an M1-dominant profile, increasing oxidative stress and inflammatory injury. ROS impair nitric oxide signaling, reduce vascular compliance, and contribute to endothelial dysfunction, while oxidative stress disrupts the coordinated uNK–macrophage interactions required for spiral artery remodeling and immune tolerance [33].

### 3.2. Preeclampsia

Preeclampsia is a pregnancy-specific hypertensive disorder that typically arises after 20 weeks of gestation and is characterized by new-onset hypertension accompanied by proteinuria and/or evidence of maternal organ or uteroplacental dysfunction [1,2,34]. Notably, some affected women may exhibit the clinical features of preeclampsia in the absence of proteinuria [35]. Globally, preeclampsia complicates approximately 3–8% of pregnancies, with substantial geographic and demographic variability in prevalence [3].

Clinically, preeclampsia is classified into early-onset (<34 weeks) and late-onset (≥34 weeks) forms, which differ in pathophysiology, disease progression, and outcomes [36]. Early-onset preeclampsia, accounting for roughly 10% of cases, is primarily driven by abnormal placentation, impaired spiral artery remodeling, and reduced uteroplacental perfusion and is frequently associated with fetal growth restriction and adverse maternal and perinatal outcomes [14]. In contrast, late-onset preeclampsia comprises approximately 90% of cases and is more closely linked to maternal cardiovascular and metabolic vulnerability, typically presenting with milder clinical manifestations and more favorable outcomes [37].

Multiple maternal risk factors for preeclampsia have been identified, including chronic hypertension, autoimmune disorders such as systemic lupus erythematosus, chronic kidney disease, nulliparity, a history of stillbirth, pregestational diabetes, elevated body mass index, prior preeclampsia, and advanced maternal age [1,2,38]. The risk of recurrence in subsequent pregnancies ranges from 25% to 67%, depending on the severity and gestational age at the onset of the initial episode [39,40,41,42].

The oxidative stress hypothesis of preeclampsia originated in the 1980s following reports of increased lipid peroxidation in affected women, including elevated malondialdehyde (MDA) levels in maternal plasma and placental tissue, which is indicative of systemic oxidative injury [43]. Subsequent observations of reduced antioxidant enzyme activity and depletion of non-enzymatic antioxidants further substantiated impaired redox homeostasis in preeclampsia [44]. These findings were incorporated into the two-stage model of pathogenesis, wherein abnormal placentation and defective spiral artery remodeling (stage 1) result in placental hypoxia and ischemia–reperfusion injury, excessive ROS production, and the release of pathogenic factors into the maternal circulation, ultimately leading to the maternal syndrome (stage 2) characterized by systemic endothelial dysfunction, inflammation, and multisystem involvement [10,45,46].

### 3.3. Oxidative Stress in Normal Pregnancy and Preeclampsia

**Figure 3:**
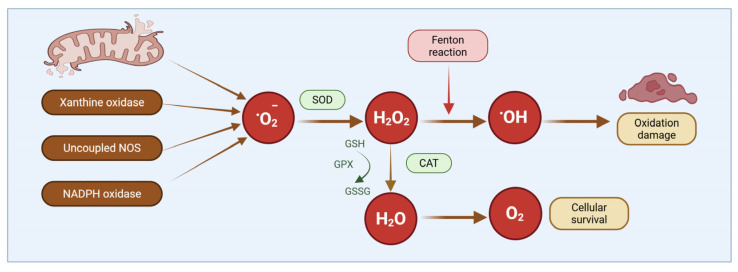
*Sources of reactive oxygen species (ROS):* ROS arise from multiple sources, primarily mitochondria, xanthine oxidase (XO), NADPH oxidases (NOXs), and uncoupled nitric oxide synthase (NOS). Superoxides (O_2_^•−^) are subsequently converted to secondary ROS, including hydrogen peroxide (H_2_O_2_), which can generate hydroxyl radicals (HO^•^) via Fe^2+^-catalyzed Fenton reactions. To limit oxidative injury, superoxides and their derivatives are detoxified by endogenous antioxidant systems, primarily superoxide dismutase (SOD), catalase (CAT), glutathione peroxidase (GPx), and the non-enzymatic antioxidant glutathione (GSH). Created in Biorender. Theera Tongsong (2026), https://app.biorender.com/illustrations/698fd08182d9d3bd9b994390 (accessed on 27 February 2026).

In normal pregnancy, tightly controlled low-to-moderate levels of ROS, generated primarily from mitochondrial respiration and NADPH oxidase (NOX) isoforms (NOX1 and NOX4), function as essential signaling mediators [23,47] (Figure 3). The main sources of ROS are summarized in Table 1. These species activate redox-sensitive kinases and transcription factors, including nuclear factor erythroid 2-related factor 2 (NRF2) and hypoxia-inducible factor-1α (HIF-1α), thereby augmenting VEGF signaling, promoting vascular smooth muscle cell phenotypic modulation, and facilitating MMP activation. Collectively, these mechanisms support placental angiogenesis and physiological vascular remodeling. Pathological oxidative stress arises when the production of reactive oxygen and nitrogen species (ROS/RNS) exceeds antioxidant capacity, disrupting redox homeostasis. Although ROS/RNS are indispensable for normal cellular signaling, their excessive accumulation, commonly triggered by placental ischemia–reperfusion injury, induces nonspecific oxidative damage to lipids, proteins, and nucleic acids, culminating in cellular dysfunction or death. Antioxidant defenses comprise enzymatic systems, including superoxide dismutase, catalase, glutathione peroxidase (GPx), and thioredoxin reductase, as well as non-enzymatic antioxidants such as glutathione, vitamins C and E, and uric acid [48,49,50]. NRF2 acts as the central regulator of antioxidant gene expression, orchestrating adaptive responses to oxidative stress [51]. Excess ROS/RNS promote lipid peroxidation, generating reactive aldehydes such as malondialdehyde and 4-hydroxynonenal; induce oxidative protein modifications; and cause DNA lesions, including 8-hydroxy-2′-deoxyguanosine lesions [52,53,54].

Mechanistically, redox imbalance in preeclampsia, driven by enhanced NADPH oxidase activity, mitochondrial dysfunction, and xanthine oxidase activation, reduces nitric oxide bioavailability, promotes endothelial nitric oxide synthase uncoupling, and increases peroxynitrite formation, thereby contributing to endothelial dysfunction, aberrant extracellular matrix deposition, and vascular stiffening [65,66]. Accumulating evidence positions oxidative stress as a central integrator of placental mitochondrial dysfunction, inflammatory activation, and angiogenic imbalance. Dysregulation of key pathways, including Nrf2/HO-1, NF-κB, PI3K/Akt, and HIF-1α, together with elevated expression of biomarkers such as MDA, 8-isoprostane, and the sFlt-1/PlGF ratio, characterizes the oxidative milieu underlying disease progression. More recent data highlight trophoblastic mitochondrial dysfunction, manifested by impaired electron transport chain activity, reduced ATP generation, and increased mitochondrial ROS production, as a principal source of oxidative stress, alongside contributions from NOX2 and NOX4 isoforms [56,67,68,69]. The intricate crosstalk between oxidative, inflammatory, angiogenic, and metabolic pathways further refines this paradigm: ROS-mediated NF-κB activation enhances proinflammatory cytokine production; oxidative disruption of angiogenic signaling contributes to the sFlt-1/PlGF imbalance; and oxidative injury to eNOS exacerbates vasoconstriction and hypertension [64,70,71,72].

ROS reduce nitric oxide (NO^•^) bioavailability in preeclampsia through several interrelated mechanisms. First, excess ROS, particularly superoxides (O_2_^•−^), rapidly react with NO^•^ to generate peroxynitrite (ONOO^−^), a highly reactive oxidant [73], which further facilitates oxidative injury by inducing lipid peroxidation and promoting the formation of malondialdehyde (MDA) and related adducts [74]. Second, upregulation of arginase increases the conversion of L-arginine to L-ornithine and urea [75], thereby limiting the availability of L-arginine as a substrate for nitric oxide synthase (NOS) and consequently reducing NO^•^ production [76,77]. Third, elevated concentrations of asymmetric dimethylarginine (ADMA), an endogenous inhibitor of eNOS, which is increased in preeclampsia, directly impair enzymatic NO^•^ synthesis [7].

In brief, in normal pregnancy, extensive remodeling of the uterine spiral arteries is essential for the establishment of an adequate fetoplacental unit. Failure of this process results in placental ischemia and subsequent hypoxia–reperfusion injury, leading to excessive generation of ROS and oxidative stress. This oxidative milieu promotes inflammation, angiogenic imbalance, and cellular senescence within the placenta. Progressive placental oxidative stress further stimulates the release of soluble oxidative and inflammatory mediators into the maternal circulation, culminating in systemic oxidative stress, vascular inflammation, and endothelial dysfunction, which underly the clinical manifestations of preeclampsia. Although defective spiral artery remodeling is classically regarded as the initiating event in the pathogenesis of preeclampsia, accumulating evidence suggests that chronic placental hypoxia, irrespective of its origin, may similarly trigger oxidative stress and the downstream cascade leading to the disorder. For instance, in fetal anemia secondary to parvovirus B19 infection, placental anemic hypoxia may precipitate preeclampsia or mirror syndrome despite previously normal placental development [78]. Thus, chronic placental ischemia from diverse etiologies not limited to abnormal vascular remodeling represents a central source of oxidative stress driving the development of preeclampsia.

## 4. Molecular Mechanisms of Oxidative Stress in Preeclampsia

### 4.1. Mitochondrial Dysfunction and ROS Generation

Mitochondria are a major intracellular source of ROS, which are predominantly generated at complexes I and III of the electron transport chain through electron leakage and superoxide formation and subsequently converted to hydrogen peroxide by mitochondrial superoxide dismutase (MnSOD) [79,80]. Under physiological conditions, mitochondrial ROS (mtROS) act as essential signaling mediators regulating uterine vascular remodeling by modulating matrix metalloproteinase activity, particularly MMP-2 and MMP-9, thereby facilitating extracellular matrix degradation and adaptive increases in uteroplacental blood flow [81]. In contrast, chronic hypoxia or excessive mtROS production induces pathological oxidative stress characterized by impaired uterine artery remodeling, enhanced vasoconstriction, arterial wall thickening, and increased vascular stiffness [56,82,83,84]. These vascular changes are accompanied by mitochondrial ultrastructural abnormalities, including swelling and cristae disruption, and functional impairments such as reduced ATP synthesis, loss of mitochondrial membrane potential, and further ROS overproduction, establishing a self-amplifying cycle of mitochondrial dysfunction [85,86]. Sustained mtROS overexpression promotes oxidative damage to lipids, proteins, and mitochondrial DNA (mtDNA); depletes mitochondrial antioxidant defenses including MnSOD and GPx; and is associated with reduced activity of electron transport chain complexes I, III, and IV in preeclamptic placentas [87,88]. Due to its proximity to ROS generation sites and limited repair capacity, mtDNA is particularly vulnerable to oxidative injury, with increased oxidative base lesions and strand breaks further exacerbating mitochondrial dysfunction and ROS production [89,90,91]. In addition, the release of cell-free mtDNA from injured placental cells into the maternal circulation may act as damage-associated molecular patterns, contributing to systemic inflammation [92]. Emerging evidence also implicates ferroptosis, an iron-dependent form of regulated cell death driven by lipid peroxidation, under placental oxidative stress, as trophoblasts from preeclamptic placentas exhibit increased lipid peroxidation, reduced glutathione peroxidase 4 expression, elevated labile iron levels, and NOX2-mediated activation of the STAT3/GPX4 signaling pathway, collectively promoting trophoblast dysfunction [93,94,95].

### 4.2. Placental Hypoxia and Ischemia–Reperfusion Injury

**Figure 4:**
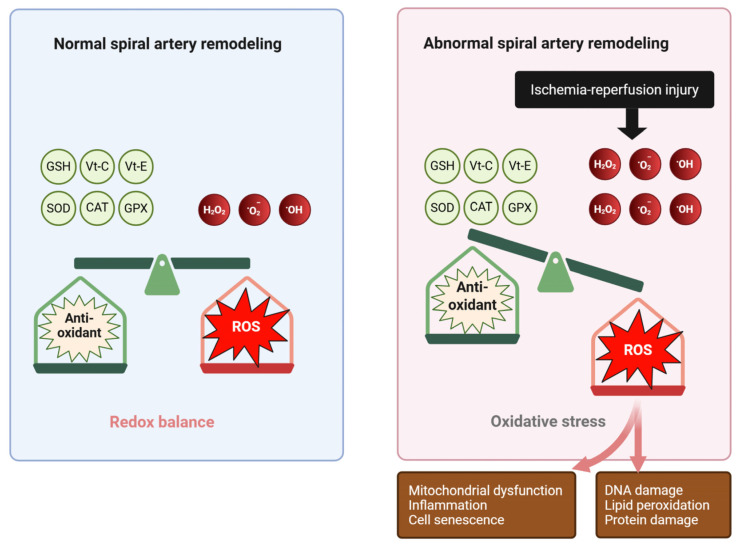
*Redox balance in placental function:* Reactive oxygen species (ROS), including hydrogen peroxide (H_2_O_2_), superoxides (O_2_^•−^), and hydroxyl radicals (HO^•^), exert beneficial effects when maintained at physiological levels (oxidative eustress), where they activate repair pathways and adaptive responses. Excessive ROS generation, however, results in oxidative stress and cellular injury. Redox homeostasis is preserved by coordinated antioxidant defenses that comprise enzymatic systems, including superoxide dismutase (SOD), catalase (CAT), and glutathione peroxidase (GPx), and non-enzymatic antioxidants such as glutathione (GSH) and vitamins C (Vt-C) and E (Vt-E). (**Left**) *Normal placenta:* Balanced ROS levels support physiological uterine vascular remodeling. (**Right**) *Preeclamptic placenta:* Ischemia and/or impaired antioxidant capacity disrupt redox equilibrium, leading to pathological oxidative damage. Created in Biorender. Theera Tongsong (2026), https://app.biorender.com/illustrations/698fd08182d9d3bd9b994390 (accessed on 27 February 2026).

In normal pregnancy, invasion of cytotrophoblasts into the uterine spiral arteries drives their transformation from high-resistance, low-capacitance vessels into low-resistance conduits capable of sustaining adequate placental perfusion [96]. Preeclampsia, by contrast, is marked by shallow trophoblast invasion and incomplete spiral artery remodeling, resulting in persistently elevated vascular resistance and restricted uteroplacental blood flow [97,98]. This defective placentation leads to placental hypoxia, which is widely regarded as an early and central event in disease pathogenesis (Figure 4).

Physiological placental hypoxia elicits adaptive responses mediated by hypoxia-inducible factors (HIFs), which coordinate transcriptional programs governing angiogenesis, metabolism, and cell survival [99,100]. In preeclampsia, however, sustained hypoxia results in excessive stabilization of HIF-1α, driving dysregulated angiogenic signaling through increased expression of soluble fms-like tyrosine kinase-1 (sFlt-1). Elevated sFlt-1 expression sequesters VEGF and PlGF, thereby disrupting angiogenic balance and contributing to placental and systemic endothelial dysfunction [101,102].

Impaired spiral artery remodeling also promotes intermittent placental perfusion, giving rise to recurrent cycles of ischemia and reperfusion that exacerbate oxidative injury [61,103,104,105,106]. During ischemia, xanthine dehydrogenase is converted to xanthine oxidase, which generates superoxide upon reperfusion, while reoxygenation simultaneously enhances mitochondrial ROS production through restoration of electron transport chain activity [107,108]. The resulting burst of ROS overwhelms placental antioxidant defenses, leading to oxidative damage, with rapid increases in superoxide levels observed within seconds to minutes following reoxygenation [109,110,111,112,113]. Repeated hypoxia–reoxygenation episodes within the intervillous space thus initiate a sustained ischemia–reperfusion-type inflammatory injury characteristic of preeclamptic placentas [106,114,115,116].

Beyond mitochondrial sources, placental hypoxia activates nicotinamide adenine dinucleotide phosphate (NADPH) oxidases, particularly NOX2 and NOX4, which are highly expressed in trophoblasts and placental endothelial cells [117]. NOX-derived ROS amplify inflammatory signaling, impair endothelial function, and disrupt normal trophoblast invasion, further aggravating placental dysfunction [57]. Consistent with a causal role, both genetic ablation and pharmacological inhibition of NOX isoforms reduce oxidative stress and ameliorate preeclampsia-like features in experimental models [118].

Additionally, placental hypoxia and oxidative stress activate endoplasmic reticulum (ER) stress and drive unfolded protein response (UPR) activation, further amplifying oxidative stress and inflammatory signaling through protein disulfide isomerase and ER oxidoreductin 1 [105,119,120]. Increased expression of ER stress markers, including glucose-regulated protein 78 (GRP78) and C/EBP homologous protein (CHOP), has been consistently observed in preeclamptic placentas, where ER stress, inflammation and apoptosis contribute to progressive placental failure [121,122]. The crosstalk between hypoxia, oxidative stress, ER stress, and inflammation creates a self-perpetuating cycle that exacerbates placental dysfunction and contributes to disease progression [6]. ER stress contributes to preeclampsia by disrupting normal placental development and triggering the release of harmful placental signals into the maternal circulation. Chronic placental stressors such as hypoxia, oxidative stress, and poor uteroplacental perfusion overwhelm the ER in trophoblast cells, leading to a maladaptive unfolded protein response that impairs trophoblast invasion, promotes apoptosis, and results in abnormal spiral artery remodeling. Persistently ER-stressed placental cells release excess anti-angiogenic and pro-inflammatory factors (notably sFlt-1 and soluble endoglin) that cause widespread maternal endothelial dysfunction [123,124,125].

### 4.3. Vascular Oxidative Damage

Endothelial dysfunction is a hallmark of preeclampsia, manifesting as impaired endothelium-dependent vasodilation, increased vascular permeability, and a prothrombotic phenotype [34,66,67]. Accumulating evidence implicates oxidative stress as a central driver of this dysfunction, acting through interrelated mechanisms that include eNOS uncoupling, diminished nitric oxide (NO) bioavailability, and direct oxidative injury to endothelial cells [9,10,16,36,67]. In preeclampsia, oxidative depletion of the essential eNOS cofactor tetrahydrobiopterin (BH4) to dihydrobiopterin (BH2) induces eNOS uncoupling, shifting enzymatic activity toward superoxide generation rather than NO synthesis [66,126,127], further reducing NO bioavailability and exacerbating oxidative and nitrosative endothelial damage [128]. Consistent with these mechanisms, reduced eNOS expression and activity in placental and maternal vascular tissues, together with elevated expression of circulating asymmetric dimethylarginine, an endogenous eNOS inhibitor, have been documented in preeclamptic pregnancies [129,130,131,132], collectively favoring sustained vasoconstriction and hypertension.

Beyond NO dysregulation, oxidative stress compromises endothelial integrity through lipid peroxidation, generating reactive aldehydes and isoprostanes that modify proteins and disrupt cellular function [129,133,134]. In particular, increased levels of 8-isoprostane, a potent vasoconstrictor and platelet activator, correlate with disease severity and further amplify vascular dysfunction in preeclampsia. Oxidative injury also promotes degradation of the endothelial glycocalyx, a key regulator of vascular permeability and mechanotransduction, leading to enhanced leukocyte adhesion and barrier disruption, as reflected by elevated expression of circulating markers of glycocalyx shedding such as syndecan-1 and hyaluronan [9,135,136].

More recently, endothelial pyroptosis has been implicated in preeclampsia-related vascular injury, with oxidative stress-driven activation of the NLRP3 inflammasome triggering caspase-1 activation, gasdermin D cleavage, and release of interleukin-1β and interleukin-18, thereby amplifying endothelial damage and systemic inflammation [11,70,88]. Pyroptosis is a caspase-dependent form of programmed cell death characterized by DNA damage, chromatin condensation, cellular swelling, membrane rupture, and the release of pro-inflammatory mediators. Unlike apoptosis, which is typically non-inflammatory, pyroptosis promotes inflammatory signaling. GSDME, a gasdermin family protein highly expressed in trophoblasts, can be cleaved by caspase-3 (CASP3) to convert CASP3-mediated apoptosis into pyroptosis, leading to the release of inflammatory cytokines such as IL-1β and IL-18. Evidence from EOPE placentas indicates that CASP3 activation and GSDME cleavage contribute to trophoblast pyroptosis and placental injury [137], consistent with observations in animal models [138,139]. In GSDME-high trophoblasts, apoptotic signaling may shift toward CASP3-dependent pyroptosis, which promotes pro-inflammatory macrophage polarization and establishes a feed-forward loop that amplifies trophoblast pyroptosis and inflammation within trophoblast–macrophage assembloids. Together, these findings suggest that CASP3-GSDME-mediated pyroptosis links apoptosis with inflammation in EOPE, highlighting CASP3 as a potential predictive biomarker and the CASP3–GSDME pathway as a promising therapeutic target for preeclampsia prevention.

### 4.4. Inflammatory Pathways and Oxidative Stress Crosstalk

**Figure 5:**
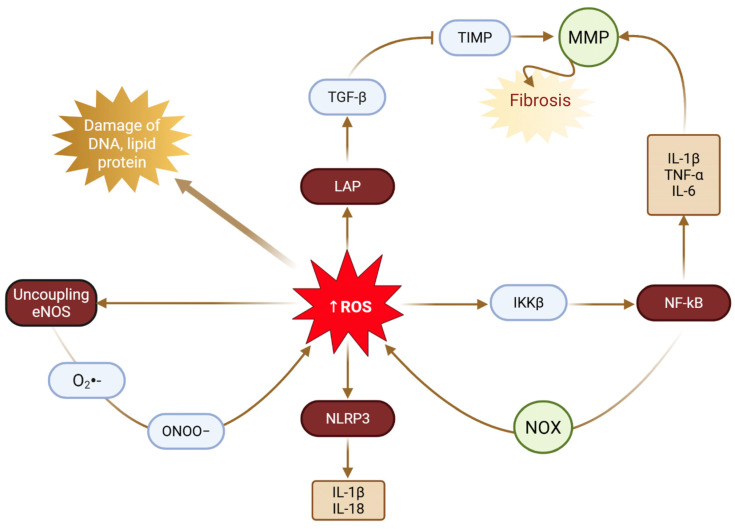
*Inflammatory pathways and oxidative stress crosstalk* in the uterine artery during pregnancy. This schematic depicts feed-forward loops of increased ROS and pro-inflammatory cytokine expression that amplifies oxidative stress and inflammation, culminating in endothelial dysfunction and defective spiral artery remodeling in preeclampsia. ROS activate NF-kappa B signaling through IκB kinase β (IKKβ), thereby upregulating the expression of TNF-α, IL-6, IL-1β, and NADPH oxidase (NOX). Enhanced NOX activity generates additional ROS, reinforcing the inflammatory cascade. Concurrently, ROS oxidize the endothelial nitric oxide synthase (eNOS) cofactor tetrahydrobiopterin (BH4) to dihydrobiopterin (BH2), leading to eNOS uncoupling and superoxide (O_2_^•−^) production. Superoxides react with nitric oxide (NO) to form peroxynitrite (ONOO^−^), promoting endothelial dysfunction. ROS also promote the release of active TGF-β from its latency-associated peptide (LAP), thereby enhancing profibrotic signaling. TGF-β contributes to fibrosis by inhibiting tissue inhibitors of metalloproteinases (TIMPs), thereby activating matrix metalloproteinases (MMPs), a process further potentiated by IL-1β and TNF-α, and driving vascular smooth muscle cells toward a fibroproliferative phenotype. The MMP/TIMP imbalance ultimately results in vascular fibrosis and impaired arterial remodeling. Created in Biorender. Theera Tongsong (2026), https://app.biorender.com/illustrations/698fd08182d9d3bd9b994390 (accessed on 27 February 2026).

Oxidative stress and inflammation are intimately linked in the pathophysiology of preeclampsia, forming a self-perpetuating cycle in which ROS activate inflammatory signaling pathways, while inflammatory mediators further amplify ROS production [11,12,13,140] (Figure 5). This reciprocal interaction operates across multiple regulatory levels, including transcriptional control, inflammasome activation, and immune cell recruitment.

Oxidative stress activates NF-κB, a master regulator of inflammatory gene expression, through ROS-mediated degradation of its inhibitory protein (IκB), thereby permitting NF-κB nuclear translocation [15,16,141,142]. NF-κB activation induces the expression of pro-inflammatory cytokines such as TNF-α, IL-1β, and IL-6, as well as adhesion molecules including ICAM-1 and VCAM-1, all of which are elevated in preeclampsia and contribute to endothelial dysfunction and systemic inflammation [12,13,67,137,143]. The inflammatory responses associated with NF-κB are summarized in Table 2.

In parallel, oxidative stress promotes activation of the NLRP3 inflammasome, a multiprotein complex that governs caspase-1 activation and the maturation of IL-1β and IL-18 [70,144,145,146]. ROS facilitate NLRP3 assembly through mechanisms involving mitochondrial dysfunction, lysosomal destabilization, and thioredoxin-interacting protein (TXNIP) dissociation [11]. Enhanced NLRP3 inflammasome activity has been documented in placental tissues and circulating immune cells from women with preeclampsia [147,148], while pharmacological inhibition of this pathway reduces inflammation and ameliorates disease manifestations in experimental models [70].

Oxidative stress-induced damage-associated molecular patterns (DAMPs) further amplify inflammatory signaling by activating toll-like receptors (TLRs), particularly TLR2 and TLR4, on maternal immune cells and endothelial cells [94,149]. Cell-free fetal DNA, mitochondrial DNA, and oxidized lipids released from stressed placental cells engage TLR signaling pathways, leading to the activation of NF-κB and interferon regulatory factors and further propagation of inflammatory responses [91,94]. Emerging evidence also indicates that TLR2/4 signaling stimulates NOX2 and NOX4 activity, thereby augmenting ROS production and promoting ferroptosis in trophoblasts [94].

Activated immune cells represent an additional source of oxidative stress in preeclampsia. Neutrophils, monocytes, and T lymphocytes from affected women exhibit heightened ROS generation and cytokine secretion [12,128]. Moreover, increased formation of neutrophil extracellular traps contributes to endothelial injury and thrombosis; this process is ROS-dependent and further intensifies oxidative stress through myeloperoxidase-mediated reactions [12,128].

**Table 2:** Some inflammatory factors contributing to endothelial dysfunction in the development of preeclampsia.

Markers	Biological Activity
Interleukin-1β (IL-1β) [6,64,150]	Mainly produced by macrophages, monocytes, and activated endothelial cells during inflammation, activating systemic inflammation through the activation of COX-2, eNOS, and endothelial adhesion molecules, such as ICAM-1 and VCAM-1.
Interleukin-2 (IL-2) [150,151]	Produced predominantly by CD4^+^ T lymphocytes and signaling cascades that promote the growth, proliferation, and differentiation of T and B lymphocytes. Stimulating the production of interferon-γ (IFN-γ) and lymphotoxin-α, thereby enhancing the activation of monocytes, neutrophils, and natural killer cells.
Interleukin-6 (IL-6) [6,64]	Produced by macrophages, monocytes, eosinophils, hepatocytes, and glial cells, with TNF-α and IL-1 serving as potent inducers of its expression. Promoting neutrophil activation and differentiation of T lymphocytes and natural killer cells.
Interleukin-8 (IL-8) [152]	Inducing cytoskeletal reorganization, changes in intracellular Ca2+ levels, integrin activation, granular protein exocytosis, and respiratory burst.
TNF-α [6,64,153,154]	Contributing to insulin resistance, influencing diverse cellular processes, playing as a central role in the inflammatory response by participating in the cytokine cascade and stimulating the production of additional pro-inflammatory mediators.
NF-κB [6,64,155]	Activated by multiple secondary messenger pathways, leading to the transcriptional upregulation of proinflammatory genes, particularly proinflammatory cytokines such as tumor necrosis factor-α (TNF-α) and interleukin-1 (IL-1).

The principal signaling pathways mediating oxidative stress–inflammation crosstalk in preeclampsia include

• Nrf2/Keap1/HO-1 axis: A key regulator of antioxidant defense. Oxidative stress triggers Nrf2 nuclear translocation and activation of antioxidant genes [51,156,157,158,159,160,161], while HO-1 exerts antioxidant, anti-inflammatory, and vasodilatory effects [51]. In preeclampsia, reduced Nrf2 activity reflects impaired antioxidant responses [156,157,158,159,160,161]; several pharmacologic and natural Nrf2 activators have shown protective effects in experimental models [51,57,157,160,161,162].
• NF-κB signaling: A central pathway regulating the inflammatory responses and trophoblast–immune cell interactions required for spiral artery remodeling [11,64,67,70]. Aberrant activation in preeclampsia promotes uteroplacental dysfunction, systemic inflammation, and endothelial injury, while oxidative stress further amplifies NF-κB signaling, forming a self-reinforcing inflammatory–oxidative loop [11,12,13,70,142,163].
• PI3K/Akt pathway: A critical regulator of cell survival, proliferation, angiogenesis, and antioxidant capacity [107,164]. Impaired signaling in preeclampsia contributes to trophoblast apoptosis and defective invasion [107,109,117]. Restoration of PI3K/Akt activity alleviates oxidative stress and disease features in experimental models [107,109,117].
• HIF-1α hypoxia response: HIF-1α is a central mediator of cellular adaptation to hypoxia, regulating genes involved in angiogenesis, metabolism, and survival [101,146,165]. Persistent placental hypoxia leads to excessive HIF-1α stabilization, particularly in early-onset preeclampsia [14,108,146]. While transient activation is adaptive, sustained activation promotes anti-angiogenic factor production (e.g., sFlt-1), inflammatory signaling, and placental dysfunction [71].

### 4.5. Integration of Mechanistic Pathways

**Figure 6:**
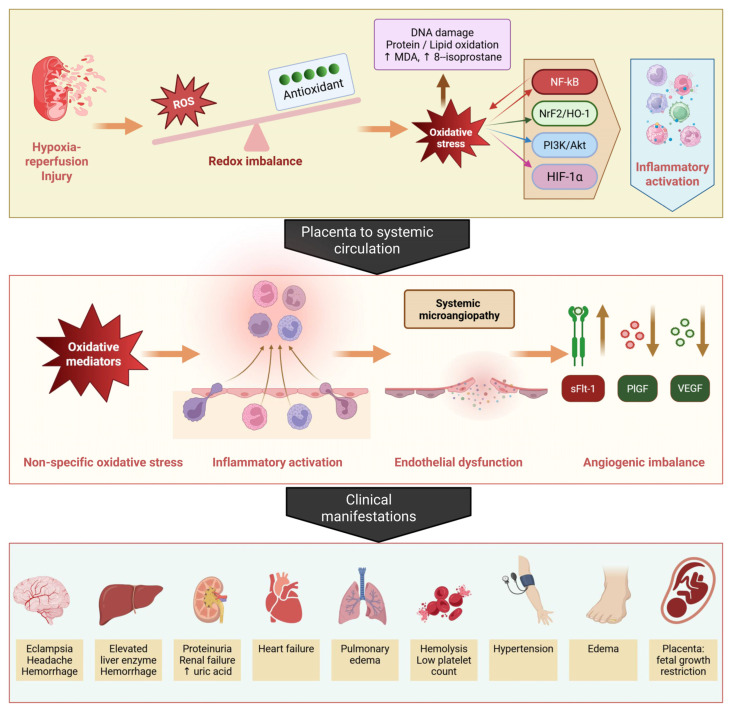
Summary of the pathophysiological progression of preeclampsia, from molecular responses to placental hypoxic injury to the development of clinical manifestations. Created in Biorender. Theera Tongsong (2026), https://app.biorender.com/illustrations/698fd08182d9d3bd9b994390 (accessed on 27 February 2026).

Evidence synthesized in this review indicates that oxidative stress in preeclampsia is not an isolated event but a complex, interconnected process involving multiple molecular pathways across cellular compartments and organ systems. This process can be conceptualized as a pathogenic cascade initiated by inadequate placentation and placental hypoxia, followed by mitochondrial dysfunction and excessive ROS generation, ultimately leading to systemic endothelial dysfunction and clinical manifestations of preeclampsia (Figure 6).

At the cellular level, mitochondrial dysfunction represents a central link between hypoxia, oxidative stress, and dysregulated angiogenic signaling. Placental hypoxia disrupts mitochondrial electron transport, increasing superoxide production and stabilizing HIF-1α, which subsequently promotes the expression of sFlt-1 and other anti-angiogenic factors. Concurrently, mitochondrial oxidative stress induces trophoblast ferroptosis and apoptosis, further impairing placental function.

The interplay among signaling pathways further increases complexity. The Nrf2/Keap1/HO-1 axis constitutes the primary antioxidant defense but is often insufficient to counteract excessive ROS in preeclampsia. In contrast, oxidative stress activates the NF-κB pathway, promoting inflammatory responses that amplify oxidative injury. Meanwhile, impairment of the PI3K/Akt pathway, which normally supports cell survival and Nrf2 activation, creates a self-perpetuating cycle of oxidative stress and cellular damage. These interconnected mechanisms help explain the limited efficacy of single-target therapies and support the potential benefit of multi-targeted therapeutic strategies.

The systemic effects of placental oxidative stress are not confined to the placenta; they also involve maternal endothelial and immune cells, ultimately affecting multiple organ systems. Factors released into the maternal circulation from the oxidatively stressed placenta, including sFlt-1, soluble endoglin, inflammatory cytokines, and oxidized lipids, promote widespread endothelial activation and dysfunction. This systemic endothelial impairment contributes to the clinical manifestations of preeclampsia, including hypertension, proteinuria, and the multi-organ complications observed in severe diseases.

In brief, the pathogenesis of preeclampsia can be summarized as follows: The central underlying mechanism is placental oxidative stress that is induced by chronic hypoxia secondary to various etiologies, most classically abnormal spiral artery remodeling. Placental oxidative mediators are released into the systemic circulation, thereby inducing inflammatory responses and systemic oxidative stress in the peripheral vascular endothelium, leading to the subsequent clinical manifestations of endothelial dysfunction, as illustrated in Figure 6.

## 5. Oxidative Stress Biomarkers in Preeclampsia

### 5.1. Lipid Peroxidation Products

Lipid peroxidation, defined as the oxidative degradation of polyunsaturated fatty acids within cellular membranes, generates reactive aldehydes that serve as established markers of oxidative stress [52]. Malondialdehyde (MDA), the most extensively studied lipid peroxidation byproduct, is consistently elevated in maternal plasma and placental tissue in preeclampsia and correlates with disease severity and adverse pregnancy outcomes [37,44]. Systematic reviews and meta-analyses confirm significantly higher MDA concentrations in women with preeclampsia compared with normotensive pregnancies, with an odds ratio of 2.37 (95% CI: 1.03, 3.70), and suggest potential diagnostic value, with elevations detectable prior to clinical disease onset [135]. Isoprostanes, prostaglandin-like compounds formed via non-enzymatic oxidation of arachidonic acid, represent more specific biomarkers of lipid peroxidation and are considered among the most reliable biomarkers of in vivo oxidative stress [8]. In particular, 8-isoprostane (8-iso-PGF2α) is markedly elevated in maternal plasma and placental tissue in preeclampsia, and longitudinal studies demonstrate a progressive rise from early gestation in women who subsequently develop the disease, supporting its potential value for early prediction [9,39,128]. These oxidative stress markers should be further explored in larger cohorts for preeclampsia diagnosis.

### 5.2. Reactive Oxygen and Nitrogen Species

Direct quantification of ROS/RNS is challenging due to their high reactivity and short half-lives; however, indirect assessment using electron paramagnetic resonance (EPR) spectroscopy, fluorescent probes, and chemiluminescence has provided valuable insights into redox imbalance in preeclampsia [49]. EPR studies demonstrate increased free radical generation in the maternal serum of women with preeclampsia, correlating with markers of endothelial dysfunction [49].

Elevated superoxide anion (O_2_^•−^) production has been detected in placental tissue, maternal leukocytes, and endothelial cells in preeclampsia, with flow cytometric analyses using dihydroethidium fluorescence confirming increased superoxide levels in circulating neutrophils and monocytes [69,128]. Excess superoxide formation promotes endothelial nitric oxide synthase uncoupling and reduces nitric oxide bioavailability; despite increased inducible nitric oxide synthase expression, NO is rapidly scavenged by superoxides to form peroxynitrite (ONOO^−^), a potent oxidant [128,166]. Peroxynitrite-mediated protein nitration generates 3-nitrotyrosine residues, which are markedly elevated in preeclamptic placental tissue and maternal circulation and serve as established biomarkers of nitrosative stress [8].

### 5.3. Antioxidant Enzyme Systems

Antioxidant defenses are compromised in preeclampsia, reflecting an overwhelmed redox system characterized by reduced activity of key enzymatic antioxidants [50]. Superoxide dismutase (SOD), which catalyzes the dismutation of superoxides to hydrogen peroxide, exhibits decreased activity in maternal erythrocytes and placental tissue, affecting both cytosolic (Cu/Zn-SOD) and mitochondrial (Mn-SOD) isoforms [44]. Catalase, which is responsible for hydrogen peroxide detoxification, is similarly reduced in preeclamptic placentas and maternal circulation, promoting hydrogen peroxide accumulation and oxidative injury; moreover, catalase gene polymorphisms have been associated with increased susceptibility to preeclampsia in certain populations [44].

Glutathione peroxidase (GPX), a selenium-dependent enzyme that reduces hydrogen peroxide and lipid hydroperoxides, is also diminished in preeclampsia [50]. In particular, reduced expression of GPX4, a key regulator of lipid peroxidation and ferroptosis, contributes to ferroptotic placental cell death and oxidative damage in affected pregnancies [94,95]. In parallel, the expression levels of glutathione (GSH), the principal non-enzymatic antioxidant, are decreased in maternal erythrocytes and placental tissue; this is accompanied by a reduced GSH/GSSG ratio, indicating impaired cellular redox homeostasis and diminished capacity to neutralize reactive species [50].

### 5.4. Angiogenic Factor Imbalance

**Figure 7:**
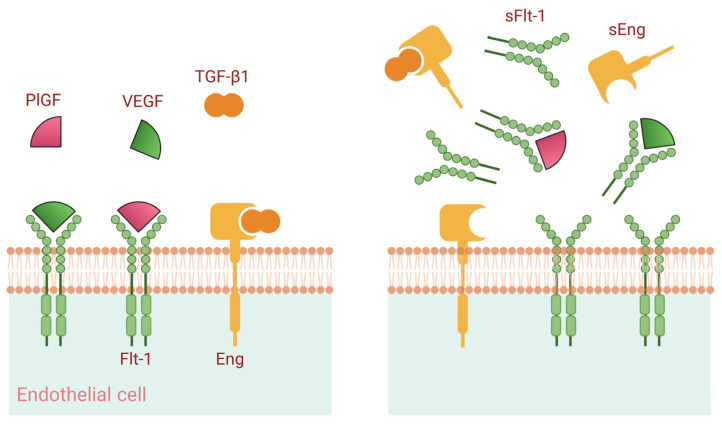
Angiogenic factor signaling in a normal pregnancy and preeclampsia: In a normal pregnancy (**left**), vascular endothelial growth factor (VEGF) and transforming growth factor-β1 (TGF-β1) maintain vascular integrity and promote vasodilation through their respective membrane receptors: fms-like tyrosine kinase-1 (Flt-1) and endoglin (Eng). In preeclampsia (**right**), excessive placental release of the soluble Flt-1 (sFlt-1) and endoglin (sEng) receptors sequesters VEGF and TGF-β1, preventing their interaction with endothelial surface receptors and thereby disrupting downstream signaling pathways. This anti-angiogenic imbalance results in endothelial dysfunction. Created in Biorender. Theera Tongsong (2026), https://app.biorender.com/illustrations/698fd08182d9d3bd9b994390 (accessed on 27 February 2026).

An imbalance between pro-angiogenic and anti-angiogenic factors is a central feature of preeclampsia, with oxidative stress contributing to this dysregulation [167,168]. Soluble fms-like tyrosine kinase-1 (sFlt-1), a truncated splice variant of the vascular endothelial growth factor receptor lacking transmembrane and intracellular domains, is markedly elevated in preeclampsia and sequesters vascular endothelial growth factor and PlGF, thereby impairing endothelial signaling and promoting endothelial dysfunction [167,168]. VEGF and TGF-β1 are essential for maintaining endothelial integrity and vascular homeostasis in multiple tissues, including the kidney and the placenta. In normal pregnancy, physiological levels of VEGF and TGF-β1 signaling support vascular adaptation and endothelial health. In preeclampsia, excessive placental release of the circulating anti-angiogenic factors sFlt-1 and sEng disrupts VEGF- and TGF-β1-mediated pathways (Figure 7). This imbalance leads to endothelial dysfunction, characterized by reduced production of nitric oxide and prostacyclin, along with increased release of procoagulant factors. Concurrently, reduced PlGF levels result in a substantially increased sFlt-1/PlGF ratio, which rises weeks before clinical onset and correlates with disease severity, supporting its clinical utility for prediction, diagnosis, and risk stratification [71]. Emerging evidence further indicates that aspirin therapy favorably modulates the sFlt-1/PlGF ratio in parallel with reductions in oxidative stress markers, highlighting a potential mechanistic link between redox balance and angiogenic regulation in preeclampsia [71].

Soluble endoglin (sEng), another anti-angiogenic factor that is elevated in preeclampsia, acts synergistically with sFlt-1 to exacerbate endothelial dysfunction. Suppression of the sFlt-1/sENG/TNF-α signaling pathway mediated by HIF-1α enhances angiogenesis and alleviates preeclampsia, underscoring the complex interplay between hypoxia signaling, angiogenic regulation, and inflammatory pathways [101]. In a large observational study, circulating concentrations of soluble endoglin (sEng) were shown to reflect the severity of preeclampsia and were associated with an increased risk of adverse outcomes, including HELLP syndrome [169]. Additionally, maternal sEng levels were strongly correlated with the degree of placental maternal vascular malperfusion, suggesting a relationship with greater disease severity. However, these findings require further confirmation in larger cohorts [170].

In summary, while the sFlt-1/PlGF ratio has been well established for clinical use, other potential biomarkers for preeclampsia, including malondialdehyde, 8-isoprostane, and nitrotyrosine, require further evaluation in larger cohorts. Though many oxidative stress biomarkers appear to be helpful in differentiating preeclamptic patients from those with normal pregnancies, clinical studies evaluating their role in early prediction are scarce and challenging. The integration of oxidative stress biomarker monitoring with conventional approaches may enable more personalized and effective strategies for the prevention of preeclampsia. For example, combining sFlt-1/PlGF ratio monitoring with oxidative stress marker assessment to guide preventive therapy represents a step toward precision medicine in preeclampsia management [71].

Interpretation of oxidative markers during labor should be approached with caution. Labor can induce placental oxidative stress primarily through ischemia and reperfusion caused by intermittent uteroplacental perfusion during uterine contractions [171,172]. Reduced blood flow during contractions and reoxygenation during relaxation generate bursts of reactive oxygen species via xanthine oxidase activation, mitochondrial dysfunction, NADPH oxidase activity, and inflammatory cell infiltration [173,174,175].

## 6. Clinical Perspectives

Preeclampsia is clinically classified into early-onset preeclampsia (EOPE, <34 weeks) and late-onset preeclampsia (LOPE, ≥34 weeks). Although considered a spectrum of the same disorder, the two subtypes differ in clinical phenotype and pathophysiology, particularly regarding placental involvement and oxidative stress [36]. EOPE typically represents a more severe form characterized by abnormal placentation, greater placental pathology, and poorer maternal and fetal outcomes, whereas LOPE more often arises after relatively normal early placentation and generally exhibits milder clinical features and a more favorable prognosis [14,37]. Mitochondrial dysfunction is more pronounced in EOPE placentas, with greater mitochondrial ROS accumulation, ultrastructural damage, and reduced ATP production [14]. Consistently, oxidative stress markers, including malondialdehyde, 8-isoprostane, protein carbonyls, and total superoxide production, are higher in EOPE and are accompanied by greater reductions in antioxidant enzyme activity compared with LOPE [14,163]. Both subtypes show dysregulation of angiogenic factors such as sFlt-1, although elevations and sFlt-1/PlGF ratios are typically more pronounced in EOPE, indicating stronger coupling between oxidative stress and anti-angiogenic signaling [71,176,177,178]. Nevertheless, while ferroptosis-related markers increase in both forms, LOPE demonstrates particularly marked placental lipid peroxidation, suggesting subtype-specific oxidative injury patterns [37]. The placenta in LOPE may experience oxidative stress not from intrinsic hypoxia due to failed spiral artery remodeling, but rather from exposure to maternal oxidative and inflammatory factors or from secondary hemodynamic stress as maternal cardiovascular adaptation fails [104].

Taken together, these findings suggest that EOPE and LOPE may share a common underlying mechanism related to placental hypoxia but differ in the degree of placental dysfunction and the contribution of maternal susceptibility or different primary causes of placental hypoxia. While EOPE is associated with abnormal placental remodeling in early gestation, placentation in LOPE is often relatively normal during early gestation, with oxidative stress emerging later in pregnancy. LOPE may reflect relatively mild placental hypoxia superimposed onto maternal cardiovascular or metabolic disorders, in which pre-existing vascular dysfunction increases vulnerability to placental hypoxia-induced oxidative stress. Consequently, a modest degree of placental oxidative stress may be insufficient to induce clinical preeclampsia in otherwise healthy women but may precipitate the disorder in those with underlying metabolic or cardiovascular conditions due to heightened vascular inflammation and endothelial dysfunction.

Nevertheless, LOPE is not invariably mild. For instance, mirror syndrome, a variant of preeclampsia characterized by maternal hypertension, proteinuria, edema, and fetal hydrops secondary to fetal anemia, can result in severe maternal disease despite previously normal placentation. Fetal hydrops due to anemia, particularly fetal alpha-thalassemia, is strongly associated with preeclampsia [179,180,181,182]. Fetal anemic hypoxia may induce placental hypoxia and oxidative mediator release into the maternal circulation even before clinical manifestations [183]. Elevated sFlt-1 levels and preeclampsia have also been reported in pregnancies with hydrops fetalis caused by parvovirus B19 infection [184]. Moreover, hydropic villi can narrow the intervillous space, further impairing placental perfusion and aggravating hypoxia [185]. Notably, preeclampsia related to fetal hydrops from anemia often resolves after correction of fetal anemia by intrauterine transfusion or after spontaneous resolution of the underlying cause, likely through alleviation of placental hypoxia [78,186,187,188,189,190,191]. These observations suggest that, in selected cases, preeclampsia may be treatable and that delivery may not always be the only definitive cure.

Clinically, EOPE can be prevented with low-dose aspirin, whereas LOPE currently lacks a well-established preventive strategy. Theoretically, LOPE may be mitigated through lifestyle modifications, such as optimizing metabolic and cardiovascular health, or through intrauterine management of fetal anemia to prevent hydrops fetalis. However, further studies are required to confirm the effectiveness of these potential preventive approaches.

## 7. Therapeutic Interventions

### 7.1. Natural Antioxidants and Dietary Supplements

Although naturally occurring antioxidants such as vitamins C, D, and E have been hypothesized to mitigate oxidative stress, evidence from randomized studies, including those involving high-risk women, has not supported their clinical efficacy. Antioxidant supplementation has been extensively investigated for the prevention and treatment of preeclampsia; however, clinical outcomes have been inconsistent [9]. Early large-scale randomized trials evaluating vitamins C and E, including the Combined Antioxidant and Preeclampsia Prediction Study (CAPPS) conducted by the Maternal-Fetal Medicine Units Network, demonstrated no reduction in preeclampsia incidence and raised concerns regarding potential adverse effects [104,192,193]. More recent strategies targeting specific redox-sensitive molecular pathways have shown more promising results in preclinical models and early-phase clinical investigations, suggesting that pathway-directed interventions may offer greater therapeutic potential [9].

Coenzyme Q10 (CoQ10), a mitochondrial electron transport chain component, attenuates oxidative stress in experimental models, partly via Nrf2/HO-1 activation, and limited clinical evidence suggests potential risk reduction in high-risk women, pending confirmation in large trials [51]. Selenium, a glutathione peroxidase cofactor, may be beneficial in deficient populations, although optimal dosing and timing are unclear [50].

Vitamin D exhibits pleiotropic effects, including enhanced VEGF expression, reduced oxidative stress markers, and improved placental structure, likely through immunomodulatory mechanisms [9]. Dietary polyphenols, such as epigallocatechin gallate, luteolin, and mangiferin, exert antioxidant, anti-inflammatory, and vasodilatory effects via the eNOS/Nrf2/HO-1, NF-κB, and PI3K/Akt/mTOR pathways in preclinical models [64,109,161]. Traditional compounds, including Astragalus, Tianma Gouteng Decoction, and astaxanthin, similarly modulate oxidative stress and nitric oxide signaling in experimental preeclampsia models [160,162]. Despite promising experimental data, rigorous clinical trials are required before routine implementation [162].

Statins were proposed to prevent preeclampsia because they stimulate heme oxygenase-1 expression, which inhibits sF1t-1 release. Pravastatin mitigates stress signaling responses in hyperglycemic conditions, weakening processes that lead to abnormal cytotrophoblast migration and invasion associated with preeclampsia [194]. The MFMU Network plans a randomized trial to test pravastatin for prevention, and a pilot study showing a favorable risk–benefit analysis justifies using pravastatin in a larger clinical trial with dose escalation [195].

In summary, despite encouraging findings from preclinical studies, clinical trials evaluating antioxidant supplementation, particularly vitamins C and E, have generally failed to demonstrate efficacy in preventing preeclampsia or improving clinical outcomes [135]. This inconsistency may be attributed to factors such as limited antioxidant bioavailability, suboptimal timing of intervention, heterogeneity in patient selection, or the complex and multifactorial nature of preeclampsia pathogenesis, which may not be adequately addressed by simple antioxidant supplementation [135]. Consequently, more targeted strategies aimed at specific oxidative stress pathways may offer greater therapeutic potential [56].

### 7.2. Mitochondria-Targeted Therapies

Given the central role of mitochondrial dysfunction in preeclampsia, mitochondria-targeted antioxidants represent a rational therapeutic strategy [79]. These compounds accumulate within mitochondria via lipophilic cations or targeting peptides, achieving high local concentrations at major sites of ROS generation [196].

MitoQ, a mitochondria-accumulating ubiquinone derivative, effectively reduces mtROS production and has been shown to lower maternal blood pressure, proteinuria, and placental oxidative stress in experimental models [79,80,196]. Similarly, AP39, a mitochondria-targeted hydrogen sulfide donor, improves mitochondrial bioenergetics and attenuates oxidative injury, thereby ameliorating preeclampsia-like phenotypes in preclinical studies [167].

Although mitochondrial antioxidants have not yet been evaluated in clinical trials for preeclampsia, promising preclinical evidence supports their translational potential [56,197]. Before clinical application in pregnancy, their safety, pharmacokinetics, and feto-placental transfer must be established in animal models. Among these agents, MitoQ has been most extensively studied and appears safe in pregnant rodents, although early administration may exacerbate preeclamptic features, highlighting the importance of optimal timing. MitoTempo and ergothioneine (ERG) have also shown no maternal or fetal toxicity in animal studies, though pharmacokinetic data remain limited.

Emerging agents include organofluorine diaryl hydrazones (e.g., HY-12), which suppress mitochondrial oxidative stress and anti-angiogenic signaling in vitro [79], and α-lipoic acid, which improves maternal and placental outcomes in preclinical studies, likely through antioxidant regeneration and metal chelation [79]. In experimental models, HY-12 shows therapeutic promise through its ability to reduce trophoblast oxidative stress and enhance mitochondrial function in vitro, improving the angiogenic balance in cultured endothelial cells exposed to preeclamptic serum [86]. These characteristics make it of interest for the treatment of preeclampsia, where oxidative stress drives pathogenesis. Thus, the preliminary result supports the testing of HY-12 in a relevant in vivo model of preeclampsia. This novel compound represents a potential therapeutic lead for further development.

### 7.3. Pathway-Specific Modulators

Targeting pathways linking oxidative stress and inflammation offer a selective therapeutic strategy for preeclampsia [118].

**NOX inhibitors**: NOX inhibition reduces ROS generation and trophoblast ferroptosis in preeclampsia via STAT3/GPX4 activation [93,94,95]. Apocynin, a NOX inhibitor, improves maternal and placental outcomes in experimental models, and combined apocynin–aspirin therapy further activates PI3K/Nrf2/HO-1 signaling, ameliorating preeclampsia symptoms [57,118]. The combination therapy was more effective than either agent alone, suggesting synergistic effects. Ferroptosis inhibition represents a complementary approach. Antiferroptotic agents decrease placental injury and sFlt-1 release, while ferrostatin-1 and liproxstatin-1 protect trophoblasts from oxidative cell death. However, given the essential physiological functions of NOX enzymes, nonspecific inhibition may entail unintended risks [118].

**Nrf2 activators:** Activation of the Nrf2 signaling pathway enhances endogenous antioxidant defenses by upregulating the transcription of cytoprotective and antioxidant genes [157]. To date, direct pharmacologic Nrf2 activators were not extensively investigated; therefore, the development of targeted Nrf2 activators for the treatment of preeclampsia remains an active area of investigation. Nevertheless, several agents, like metformin [157], CoQ10 [51], and epigallocatechin gallate (EGCG) [161], appear to partly exert promising activity through the activation of this pathway. Metformin, a widely used antidiabetic medication, attenuates placental oxidative stress via Nrf2/Keap1 activation, enhancing antioxidant defenses and reducing ROS in experimental models; its preventive role in high-risk pregnancies is under clinical evaluation [157,198]. It activates AMPK, potentially improving mitochondrial function and reducing oxidative stress, and restores the angiogenic balance by decreasing sFlt-1 and sEng levels and thus has potential to prevent preeclampsia [198,199]. In a preliminary study, pre-diabetic women were given metformin or a placebo throughout their pregnancy, and metformin-treated women had a lower incidence of severe preeclampsia [200]. Given its well-established safety profile during pregnancy, metformin represents a promising candidate for further clinical evaluation in the prevention or management of preeclampsia.

Targeting inflammasome signaling also disrupts the oxidative–inflammatory feed-forward loop. The NLRP3 inhibitor MCC950 reduces inflammation and improves outcomes in preclinical models, though safety considerations remain essential before clinical translation [70].

### 7.4. Conventional Pharmacological Approaches

Several established pharmacologic agents possess antioxidant properties that may contribute to therapeutic effects in preeclampsia [9]. Low-dose aspirin, the only proven preventive intervention, reduces oxidative stress markers and favorably modulates the sFlt-1/PlGF ratio, thereby improving angiogenic balance beyond its antiplatelet action [9].

Omega-3 fatty acids, particularly eicosapentaenoic and docosahexaenoic acids, modulate inflammation. Evidence regarding their role in preeclampsia prevention is mixed: a 2018 Cochrane review found a nonsignificant risk reduction [201], whereas meta-analyses in 2020 and 2022 reported significant decreases in preeclamptic risk [202,203]. Although these findings suggest a potential protective effect, current evidence remains insufficient to recommend routine supplementation, and further large-scale randomized controlled trials are warranted.

Melatonin, a potent antioxidant and anti-inflammatory hormone, has demonstrated protective effects against endothelial pyroptosis through the activation of melatonin receptor 1 in preclinical studies. These effects are likely mediated by its antioxidant and anti-inflammatory properties. In addition, its ability to cross the placenta and its favorable safety profile support further investigation as a potential therapeutic agent [88].

### 7.5. Clinical Translation Challenges

Despite strong preclinical evidence implicating oxidative stress in preeclampsia pathogenesis, translation into effective clinical therapies has been limited [9]. The failure of vitamins C and E to confer benefit in large, randomized trials highlights the shortcomings of nonspecific antioxidant strategies [9]. Several factors likely underlie these outcomes.

First, therapeutic timing is critical, as oxidative stress emerges early in pregnancy, often preceding clinical identification of high-risk status; interventions initiated after oxidative injury is established are therefore unlikely to be effective [39]. Second, the multifactorial nature of oxidative stress limits the efficacy of single-target approaches, suggesting that combination therapies simultaneously addressing mitochondrial dysfunction, NADPH oxidase activity, and inflammatory signaling may be required, as supported by preclinical benefits observed with combined apocynin and aspirin treatment [6,57].

Third, biomarker-guided strategies may enhance therapeutic precision by identifying women most likely to benefit from antioxidant interventions. Markers such as the sFlt-1/PlGF ratio and indices of oxidative stress could facilitate patient stratification and support personalized treatment approaches [71,135]. Finally, the choice of antioxidant is pivotal; mitochondria-targeted agents that accumulate at sites of ROS generation may offer greater efficacy than systemic antioxidants, while natural compounds with pleiotropic antioxidant, anti-inflammatory, and vasodilatory properties may provide additional therapeutic advantages [79,162,196].

Some interesting studies concerning interventions targeting oxidative stress in the prevention of preeclampsia are summarized in Table 3.

## 8. Methodological Considerations and Limitations

This review has several limitations that warrant consideration [9]. Substantial heterogeneity among included studies in preeclampsia definitions, diagnostic criteria, and disease severity complicates cross-study comparisons and may obscure subtype-specific effects [34,36]. In addition, most mechanistic evidence derives from animal and in vitro models that incompletely recapitulate human preeclampsia; while informative, interspecies differences in placental structure and immune regulation constrain direct clinical translation and highlight the need for human validation studies [9]. Variability in oxidative stress biomarker assessment, including differences in methodology, biological matrices, and timing, further limits interpretability, underscoring the need for standardized assays, pregnancy-specific reference ranges, and validation in large prospective cohorts [135].

Despite these limitations, convergent evidence from molecular, biochemical, animal, and clinical studies provides strong support for the role of oxidative stress in preeclampsia pathogenesis. The consistency of findings across populations, study designs, and methodological approaches strengthens confidence in the overarching conclusions of this review [6].

## 9. Future Research Directions

This comprehensive review identifies several priorities for future research [16]. Defining the early initiating events that trigger placental oxidative stress is essential, as interindividual differences in redox homeostasis may reveal novel preventive targets [39]. Genetic and epigenetic determinants, including antioxidant enzyme variants, mitochondrial DNA polymorphisms, and epigenetic regulation of Nrf2-related pathways, likely influence susceptibility and could be leveraged through integrative multi-omics approaches to identify high-risk individuals [44,135,157]. Greater attention is also needed to the long-term maternal and offspring consequences of preeclampsia, as increased cardiovascular and metabolic risk may reflect persistent or developmentally programmed oxidative injury [3]. Promising therapeutic strategies, including mitochondria-targeted antioxidants, ferroptosis inhibitors, and pathway-specific modulators, require rigorous evaluation in pregnancy, with adaptive trial designs and biomarker-guided stratification potentially accelerating translation [79,93,118,135]. In parallel, systematic validation of oxidative stress biomarkers integrated with angiogenic factors and clinical risk profiles may improve prediction and enable point-of-care risk stratification [71,135]. Finally, delineating distinct oxidative stress mechanisms in early- versus late-onset preeclampsia may support subtype-specific interventions, particularly given the pronounced mitochondrial dysfunction observed in early-onset diseases [14,36].

## 10. Conclusions

Evidence from the literature consistently identifies oxidative stress-associated placental hypoxia as a key contributor to the development and progression of preeclampsia. An imbalance between reactive oxygen species (ROS) production and antioxidant defenses leads to placental dysfunction, lipid and protein oxidation, DNA damage, and endothelial impairment. Interacting pathways involving ROS generation, hypoxia signaling, inflammation, and endothelial nitric oxide synthase (eNOS) uncoupling promote vascular dysfunction and disrupt trophoblast invasion and spiral artery remodeling, resulting in inadequate placental perfusion and the clinical features of hypertension, proteinuria, and multi-organ involvement.

Biomarkers such as malondialdehyde (MDA), 8-iso-prostaglandin F2α, ischemia-modified albumin, and antioxidant enzymes including superoxide dismutase (SOD) and glutathione peroxidase (GPx) reflect oxidative imbalance in maternal blood and placental tissue and correlate with disease severity and adverse outcomes. However, variability in methods, timing, and biomarker specificity limits their routine clinical application.

Therapeutic studies show the limited benefit of vitamins C and E, whereas emerging agents such as melatonin, curcumin, selenium, NOX inhibitors and nutraceuticals demonstrate promising experimental effects on oxidative stress and placental–endothelial function. Differences in oxidative profiles across hypertensive pregnancy disorders suggest value in refined biomarker panels and targeted interventions.

Despite advances in mechanistic understanding, translation to practice remains challenging due to disease complexity and study heterogeneity. Future work should prioritize standardized biomarker protocols; integrated evaluation of oxidative, inflammatory, and angiogenic pathways; and rigorous testing of novel antioxidant strategies to support earlier diagnosis and more personalized management of preeclampsia.

## Figures and Tables

**Table 1:** Summary of key sources of reactive oxygen species.

ROS	Primary Sources	Key Roles
Mitochondrial ROS (superoxide) [14,55,56]	ECs, SMCs, and trophoblasts	HIF-1α/NRF2 signaling; promoting remodeling; excess levels cause stiffness
NADPH oxidases; (O_2_^•−^/H_2_O_2_) [8,47,57,58,59]	ECs, SMCs, and leukocytes	Low levels promote angiogenesis and MMP regulation; upregulated ROS; ↓ NO bioavailability
Xanthine oxidase [8,60,61]	Ecs and trophoblasts	Modest redox tone; NO regulation; excess levels cause apoptosis
Uncoupled NO Synthase [62,63]	Ecs and trophoblasts	Produces O_2_^−^ instead of NO when uncoupled; reduces NO bioavailability; contributes to endothelial dysfunction
NF-κB axis [6,64]	Multiple	Feed-forward loop to increase ROS; increasing TNF-α/IL-6/IL-1β; NOX activation

EC: endothelial cell; HIF-1α: hypoxia-inducible factor 1-alpha; NADPH: nicotinamide adenine dinucleotide phosphate; NOX: NADPH oxidase; NO: nitric oxide; NRF2: nuclear factor erythroid 2-Like 2; ROS: reactive oxygen species; SMC: smooth muscle cell; ↓: decrease.

**Table 3:** Some interventions targeting oxidative stress in the prevention of preeclampsia.

Interventions	Mechanism	Evidence-Based Results
Vitamins C and E	Free radical scavengers	Large RCTs: No benefit in prevention [192,193,204]; a concern of disrupting physiologic ROS in high-dose supplements.
Low-dose aspirin	Anti-inflammatory, improve sFlt-1/PlGF ratio, and antiplatelet effects	RCTs: Well established benefit for prevention, 20–30% risk reduction [9,205].
Selenium	A component of glutathione peroxidase	Observational studies: Lower selenium in preeclampsia. RCTs: Potentially reduced risk of preeclampsia in low-selenium groups [206,207].
Melatonin	Potent antioxidant decreases endothelial pyroptosis through melatonin receptor 1	Preclinical study and animal model: Shown to reduce OS and improve outcomes in animal models. Early-stage clinical trials ongoing [88,208].
MitoQ and SkQ1	Targeting mitochondria to reduce ROS	Preclinical models: Promising results and a potential benefit in a clinical study [80,196].
α-lipoic acid	Suppress mitochondrial ROS production	Promising results in a preclinical study [79].
NOX2 inhibitor	Ferroptosis inhibition and decrease placental sFlt-1 release	Promising results in a preclinical study [93,94,95].
Statin	Inhibit sFlt-1, targeting angiogenesis	Favorable risk–benefit trade-off in a pilot clinical study [195].
Omega-3 fatty acids	Anti-inflammatory effects	Clinical trials: The results are mixed [201,202,203]. Large-scale randomized controlled trials are warranted.
Metformin	Nrf2/Keap1 activation and reduce sFlt-1 and sEng activity	Animal model: Ameliorating preeclampsia [157], Retrospective cohort study: Lower incidence of severe preeclampsia among pre-diabetic women [200].
HY-12	reducing cell injury, mitochondrial stress, and anti-angiogenic response	Experimental model: Promising as a potential therapeutic lead for further development [86].

## Data Availability

No new data were created or analyzed in this study. Data sharing is not applicable to this article.

## Abbreviations

The following abbreviations are used in this manuscript:

Ang-1/2	Angiopoietin1/2
BH2	Dihydrobiopterin
BH4	Tetrahydrobiopterin
CAT	Catalase
COXs	Cyclooxygenases
ECM	Extracellular matrix
ECs	Endothelial cells
EDHF	Endothelium-derived hyperpolarizing factor
eNOS	Endothelial nitric oxide synthase
GPxs	Glutathione peroxidases
GSH	Glutathione
GSSG	Oxidized glutathione
H2O2	Hydrogen peroxide
HIF-1α	Hypoxia-inducible factor 1-alpha
ICAM-1	Intercellular adhesion molecule-1
IFN-γ	Interferon gamma
IKKβ	IκB kinase beta
IL-1β	Interleukin-1 beta
IL-6	Interleukin-6
IUGR	Intra-uterine growth restriction
LTB4	Leukotriene B4
MMP	Matrix-metalloproteinases
NADPH	Nicotinamide adenine dinucleotide phosphate
NF-κB	Nuclear factor-kappa B
NLRP3	NOD-like receptor family pyrin domain-containing 3
NO	Nitric oxide
NOX	NADPH oxidase
NRF2	Nuclear factor erythroid 2-Like 2
O_2_^•–^	Superoxide
ONOO^−^	Peroxynitrite
PlGF	Placental growth factor
ROS	Reactive oxygen species
sEng	Soluble endoglin
sFlt-1	Soluble fms-like tyrosine kinase-1
SMCs	Smooth muscle cells
SODs	Superoxide dismutases
TGF-β	Transforming growth factor beta
TIMP	Tissue inhibitor of metalloproteinase
TLR	Toll-like receptor
TNF-α	Tumor necrosis factor-alpha
uNK	Uterine Natural killer
VCAM-1	Vascular cell adhesion molecule-1
VEGF-A	Vascular endothelial growth factor A
XDH	Xanthine dehydrogenase
XO	Xanthine oxidase
